# Enhancement of Mitochondrial Transfer by Antioxidants in Human Mesenchymal Stem Cells

**DOI:** 10.1155/2017/8510805

**Published:** 2017-05-17

**Authors:** Chia-Jung Li, Po-Kong Chen, Li-Yi Sun, Cheng-Yoong Pang

**Affiliations:** ^1^Institute of Medical Sciences, Tzu Chi University, Hualien, Taiwan; ^2^Research Assistant Center, Show Chwan Memorial Hospital, Changhua, Taiwan; ^3^Department of Medical Research, Buddhist Tzu Chi General Hospital, Hualien, Taiwan

## Abstract

Excessive reactive oxygen species is the major component of a harsh microenvironment after ischemia/reperfusion injury in human tissues. Combined treatment of N-acetyl-L-cysteine (NAC) and L-ascorbic acid 2-phosphate (AAP) promoted the growth of human mesenchymal stem cells (hMSCs) and suppressed oxidative stress-induced cell death by enhancing mitochondrial integrity and function in vitro. In this study, we aimed to determine whether NAC and AAP (termed MCA) could enhance the therapeutic potential of hMSCs. We established a coculture system consisting of MCA-treated and H_2_O_2_-treated hMSCs and investigated the role of tunneling nanotubes (TNTs) in the exchange of mitochondria between the 2 cell populations. The consequences of mitochondria exchange were assessed by fluorescence confocal microscopy and flow cytometry. The results showed that MCA could increase the mitochondrial mass, respiratory capacity, and numbers of TNTs in hMSCs. The “energized” mitochondria were transferred to the injured hMSCs via TNTs, the oxidative stress was decreased, and the mitochondrial membrane potential of the H_2_O_2_-treated hMSCs was stabilized. The transfer of mitochondria decreased the expression of S616-phosphorylated dynamin-related protein 1, a protein that dictates the fragmentation/fission of mitochondria. Concurrently, MCA also enhanced mitophagy in the coculture system, implicating that damaged mitochondria were eliminated in order to maintain cell physiology.

## 1. Introduction

Human mesenchymal stem cells (hMSCs) are multipotent cells isolated from adult tissues and possess the potential to differentiate into various cell types [1–3]. Recently, hMSCs have been tested in rescuing tissue injuries, including ischemia/reperfusion injuries of the heart, kidney, and brain [4, 5]. Ischemia/reperfusion injuries caused complex pathology in these tissues. Among them, overproduction of reactive oxygen species (ROS) in the injured tissues usually decrease the viability of engrafted MSCs after transplantation [6]. ROS are highly reactive molecules and are derived from the metabolism of oxygen. Mitochondria are the primary sources of ROS in eukaryotic cells, and excessive ROS in mitochondria usually cause damages to mitochondria and subsequently trigger mitochondrion-mediated cell death [7, 8]. It is thus important to increase the viability of hMSCs when transplanting them into injured tissues.

Recently, a tubular and thin membranous structure named tunneling nanotubes (TNTs) have been described to serve as channels transporting intracellular components between connected cells in vitro and in vivo [9]. These cellular components range from cytoplasm, ions, lipid droplet, viral and bacterial pathogens, and organelles (including mitochondria and lysosomes as well) [10–12]. TNTs can be found in various cell types, such as vascular smooth muscle cells, endothelial cells, and MSCs and cancer cells, and have been implicated to be beneficial in the repair of injured cells/tissues by exchange of functional mitochondria through TNTs after cell therapy [9, 13, 14].

In our previous studies, we demonstrated that combined treatment of N-acetyl-L-cysteine (NAC) and L-ascorbic acid 2-phosphate (AAP) offered several advantages in human adipose-derived MSCs: (1) NAC and AAP-pretreatment promotes hMSCs proliferation and maintains its stemness; (2) NAC and AAP-pretreatment suppresses oxidative stress-induced cell death by enhancing mitochondrial integrity and functions [15, 16]. In this study, we aimed to determine whether NAC and AAP-pretreatment could enhance the therapeutic potential of hMSCs. The medium that contained both antioxidants was named mesenchymal stem cell adjuvant (MCA). We established a coculture system that consisted of MCA-treated and H_2_O_2_-treated hMSCs and investigated the formation of TNTs and the exchange of mitochondria between the 2 cell populations. The consequences of mitochondria exchange were assessed by fluorescence confocal microscopy and flow cytometry after staining the cells with mitochondrion-specific fluorescent dyes.

## 2. Materials and Methods

### 2.1. Isolation and Maintenance of Human Adipose Tissue-Derived MSCs

Adipose tissue was procured from a donor who underwent liposuction with written informed consent (Buddhist Tzu Chi General Hospital Internal Review Board# IRB102-130). The human adipocyte-derived MSCs were isolated using our previously published method [15]. The hMSCs were cultured in a medium that consisted of Iscove's modified Dulbecco's medium (IMDM; GIBCO-Invitrogen Co.), 10% FBS (MSC-Qualified, GIBCO-Invitrogen Co.), 10 ng/mL FGF-2 (R&D Systems), and 2 mM L-glutamine and adjusted to contain 0.1 M sodium bicarbonate at 37°C in a humidified incubator containing 5% CO_2_ and 95% air. All experiments were performed using hMSCs cultured between passages 3 to 6.

### 2.2. Oxygen Uptake Measurement

Mitochondrial oxygen consumption was measured using Oxygraph-2k according to the manual provided by the manufacturer (Oroboros Instruments Corp., Austria, http://wiki.oroboros.at/index.php/O2k-Protocols:_MitoPathways). The electrode was calibrated between 0 and 100% saturation with atmospheric oxygen at 37°C. In brief, respiration was measured at 37°C in 2 mL glass chambers. Cells were harvested by trypsinization, followed by centrifugation, and the cell pellet was resuspended in PBS for analysis. Maximal oxidative capacities (state 3) were determined in the presence of oxygen content of room air (21%). Respiration rates were calculated as the time derivative of oxygen measured in the closed respirometer and expressed per million viable cells. The amplified signal was recorded in a computer with online display of the calibrated oxygen concentration and oxygen flux (DatLab software for data acquisition and analysis; Oroboros Instruments, Innsbruck, Austria). Oxygen consumption is expressed as pmol O_2_/s/mg of mitochondrial protein. The reaction chamber was filled with ~1 × 10^6^ indicated cells, and the oxygen consumption rate (OCR) of each respiratory complex was measured after addition of appropriate substrates, respiratory inhibitors (2 *μ*M rotenone, 2 *μ*M antimycin A, 0.5 *μ*M oligomycin), and mitochondrial uncoupler FCCP (2 *μ*M).

### 2.3. Mitochondrial Dehydrogenase Activity Assay

Mitochondrial dehydrogenase activity was analyzed using WST-8 (2-(2-methoxy-4-nitrophenyl)-3-(4-nitrophenyl)-5-(2,4-disulfophenyl)-2H-tetrazolium; CCK-8 Cell Counting Kit-8, Enzo Sci. Inc., US) that produces a highly water soluble formazan dye upon biochemical reduction at the presence of an electron carrier, 1-methoxy-PMS. At the end of various treatments, 10 *μ*L of the CCK-8 reagent was added to each well and incubated at 37°C for additional 4 h. Absorbance at 450 nm was recorded by an ELISA microplate reader.

### 2.4. Cell Treatments

The MCA consisted of 2 mM NAC (Sigma) and 0.2 mM AAP (L-ascorbic acid 2-phosphate sesquimagnesium salt hydrate, Sigma). The cells cultured in medium without NAC and AAP were used as the normal control. Generally, for MCA protection experiments, the hMSCs were treated with MCA for 24 h and H_2_O_2_ (Sigma) for 4 h.

### 2.5. Detecting Mitochondria Transfer in Coculture System

The mitochondria of the healthy hMSCs were labeled with MitoTracker Red (Molecular Probes), and the mitochondria of the H_2_O_2_-treated hMSCs were labeled with Mitotracker Green (Molecular Probes) according to the manufacturer's instructions. To induce mitochondrial dysfunction, hMSCs were treated with 0.5 mM H_2_O_2_ for 4 h, while the healthy cells were cultured in control medium or MCA medium for 24 h. The injured cells were then cocultured at a 1 : 1 ratio with the control or MCA-treated hMSCs, respectively. Cells were observed under confocal microscopy (LSM510 Meta, Carl Zeiss) at an indicated time after coculture.

### 2.6. Quantification of Mitochondria Transfer

Healthy cells were labeled with 5-carboxyfluorescein diacetate (CFDA, Thermo Fisher Scientific) before 1 : 1 coculture with H_2_O_2_-treated hMSCs. The mitochondria of the control or MCA-treated hMSCs were stained with MitoTracker Red before 1 : 1 coculture. After 24 h coculture, all cells were harvested and subjected to flow cytometric analysis (BD FACSCalibur, US). The transfer of mitochondria was quantitated in the R2-gated CFDA-free cells. The appearance of MitoTracker Red fluorescence in the gated cells were supposed to be transferred from the control or MCA-treated hMSCs.

### 2.7. Determination of Mitochondrial Membrane Potential (Δ*Ψ*m), ROS, and Mass

Cells from each experimental condition were stained with JC-1 reagent (for Δ*Ψ*m, 5 *μ*M), MitoSOX Red (for mitochondrial ROS, 5 *μ*M), and MitoTracker Green (for mitochondrial mass, 0.1 *μ*M) (all purchased from Molecular Probes), at 37°C for 30 min. Cells were washed twice with PBS, harvested, resuspended in PBS, and subjected to cytometric analysis (BD FACSCalibur, US).

### 2.8. Western Blotting Analysis

Cells were harvested after various treatments, and the expressions of each indicated proteins were densitometrically quantitated after SDS-PAGE and immunoblotting [16].

### 2.9. Quantification of TNTs

Fluorescent staining of actin was done with phalloidin 633 (4 units/mL, Molecular Probes) in culture medium containing 0.05% Triton X. The staining was performed on live cells with 15 min incubation at 37°C prior to fixation. The number of TNTs in the actin-labelled populations was counted and expressed as the number of TNTs per 100 cells.

### 2.10. Antibodies and Inhibitors

Anti-dynamin-related protein 1 (Drp-1, #5931), anti-phospho-Drp-1 Ser616 (#53455) were purchased from Cell Signaling Technology (MA, US). The antimitofusin-2 (Mfn2) was from GeneTex (GTX102055, GeneTex Inc., US). Anti-*β*-actin (A5411) was purchased from Sigma. To inhibit gap junctions as well as microtubule polymerization, the coculture medium was loaded with 50 *μ*M nocodazole (M1404, Sigma).

### 2.11. Statistical Analysis

The intensity of bands in Western blots or fluorescence in fluorescent-micrographs were quantified using AlphaDigiDoc software (Cell Biosciences, ON, Canada), ImageJ software (NIH), and MicroP software [17]. All values are expressed as the mean ± the standard error of the mean (SEM) and were analyzed using Student's *t*-test with two-tailed distribution between groups as indicated in the figures. All calculations were performed by Microsoft Excel 2010.

## 3. Results

### 3.1. MCA Promotes Mitochondrial Biogenesis in hMSCs

**Figure 1:**
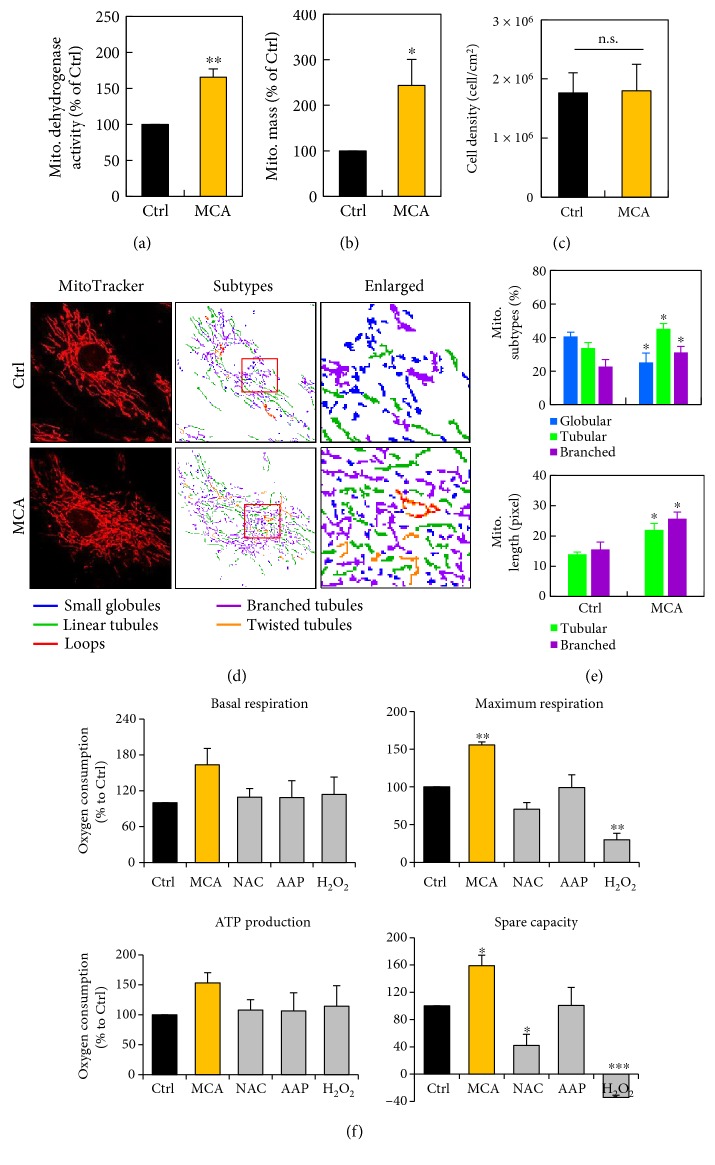
MCA improved mitochondrial biogenesis and bioenergetics in hMSCs. Mitochondrial dehydrogenase activity (a) and mitochondrial mass (b) of hMSCs treated with or without MCA for 24 h was assessed by CCK-8 assay and flow cytometric analysis of MitoTracker Red-labeled mitochondria, respectively. (c) The cell density of hMSCs with or without MCA treatment for 24 h was assessed by counting the harvested cells and normalized to the area of the culture flask. (d) The mitochondria of the hMSCs with or without MCA treatment were labeled with MitoTracker Red and subjected to confocal microscopy. The mitochondria were classified using MicroP software according to their morphology: small globules, linear tubules, loops, branched tubules, and twisted tubules. Three major types of mitochondria were quantified: globular, tubular, and branched ((e), upper panel), as well as their total length ((e), lower panel). (f) Mitochondrial bioenergetics of the indicated cells was determined by oxygen consumption rate (OCR) using Oroboros Oxygraph-2k analyzer. The MCA consisted of 2 mM NAC and 0.2 mM AAP. The concentrations of NAC and AAP were 2 mM and 0.2 mM, respectively, in monotreatments. OCR measured at basal respiration (upper left), maximal respiration (upper right), ATP-coupled respiration (lower left), and spare capacity (lower right) were shown. ^∗^*p* < 0.05, ^∗∗^*p* < 0.01, ^∗∗∗^*p* < 0.001, as compared to the control (Ctrl); n.s., not significant.

The overall mitochondrial dehydrogenase activity of hMSCs was measured at 24 h after MCA treatment. The mitochondrial dehydrogenase activity of the MCA-treated hMSCs was 65.6% higher than that of the nontreated control hMSCs (Figure 1(a)). Concurrently, the mitochondrial mass of the MCA-treated cells was 143.5% larger than the nontreated cells (Figure 1(b)). The total cell number of both groups at 24 h did not differ much (Figure 1(c)). We next observed the mitochondrial network using confocal microscopy: the mitochondria were vitally stained with fluorescent dye, MitoTracker Red. We found that the morphology of mitochondria were markedly altered after MCA treatment in hMSCs (Figure 1(d)). The changes of mitochondrial morphology were stratified with MicroP software [17]. The mitochondria were classified into five types according to the characteristics of their morphology: small globules, linear tubules, loops, branched tubules, and twisted tubules. The globular mitochondria decreased from 40.7% to 25.2%, while branched and tubular mitochondria increased from 22.7% and 33.8% to 31.1% and 45.1%, respectively. (Figure 1(e), upper panel). In addition, we also noted that the average length of the mitochondria in MCA-treated cells significantly outscored those without treatment (Figure 1(e), lower panel, linear: 22.0 pixels versus 13.9 pixels; branched: 25.7 pixels versus 15.5 pixels). The width of the mitochondria did not differ in both treatments.

The cellular bioenergetics was also evaluated in hMSCs after treatment with MCA for 24 h. Marked reduction of mitochondrial respiration was observed in H_2_O_2_-treated cells as compared to the control and MCA-treated cells (Figure 1(f)). The mitochondrial respiration efficiency of hMSCs were significantly improved after MCA treatment as compared to the control or monotreated cells (NAC or AAP alone).

### 3.2. MCA Enhances TNT Formation

**Figure 2:**
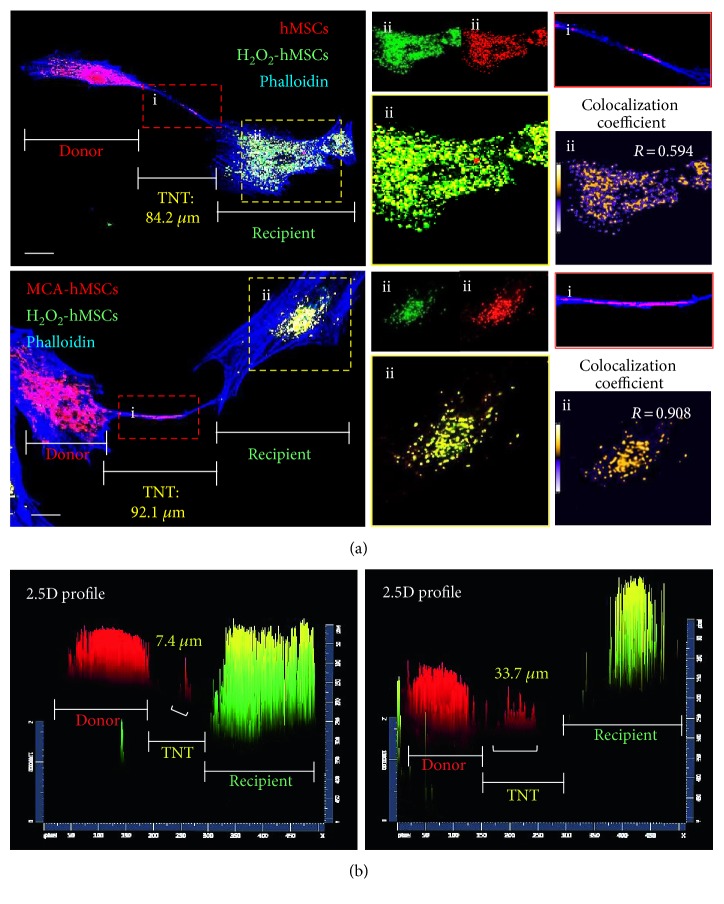
Mitochondria transfer was enhanced in hMSCs after MCA treatment. (a) Representative images of mitochondria transfer to H_2_O_2_-treated hMSCs from hMSCs with (right panel) or without (left panel) MCA treatment. The MitoTracker Red-labeled mitochondria from the healthy hMSCs were also clearly seen in TNT formed between the recipient and donor (inset i). Both MitoTracker Red- and Green-labeled mitochondria were found in the recipient cells (inset ii). The Pearson correlation coefficient are also shown to demonstrate the colocalization of the MitoTracker Red and Green labelled mitochondria after coculture [30]. The results showed that the colocalization coefficient in the (ii) of upper panel (24 h coculture of control hMSCs and H_2_O_2_-treated hMSCs) is 0.594, while that in the (ii) of lower panel (24 h coculture of MCA-treated hMSCs and H_2_O_2_-treated hMSCs) is 0.908. The colocalization coefficient can be further represented as pseudocolor changes: orange dots represent highly overlapping, while purple dots represent no overlapping. A 2.5-dimensional fluorescence reconstruction analysis of the respective images in (a) further confirmed that the red fluorescence was predominantly found in the TNT form between MCA-treated hMSCs and H_2_O_2_-treated hMSCs (b). Scale bar: 20 *μ*m.

Previous studies have shown that cellular components can be transported between two separated cells by TNTs. Cytoplasm exchange, which includes mitochondria as well, occurs when TNTs form. The mitochondria from healthy hMSCs and injured hMSCs were labeled with MitoTracker Red and MitoTracker Green, respectively. Both cells were subjected to 1 : 1 coculture. The formation of intercellular TNTs between the donor and recipient cells enabled mitochondrial exchange through the TNTs (Figure 2(a)). After 24 h coculture, most of the mitochondria were found in the recipients, that is, the H_2_O_2_-treated cells, and contained both MitoTrackers (Figure 2(a), yellow insets). Selected (*x*-*y*) sections, obtained from confocal microscopy, and 2.5-dimensional reconstructions (as represented in Figure 2(b)) allowed us to identify the transport of mitochondria as the two fluorochromes were from the same plane.

**Figure 3:**
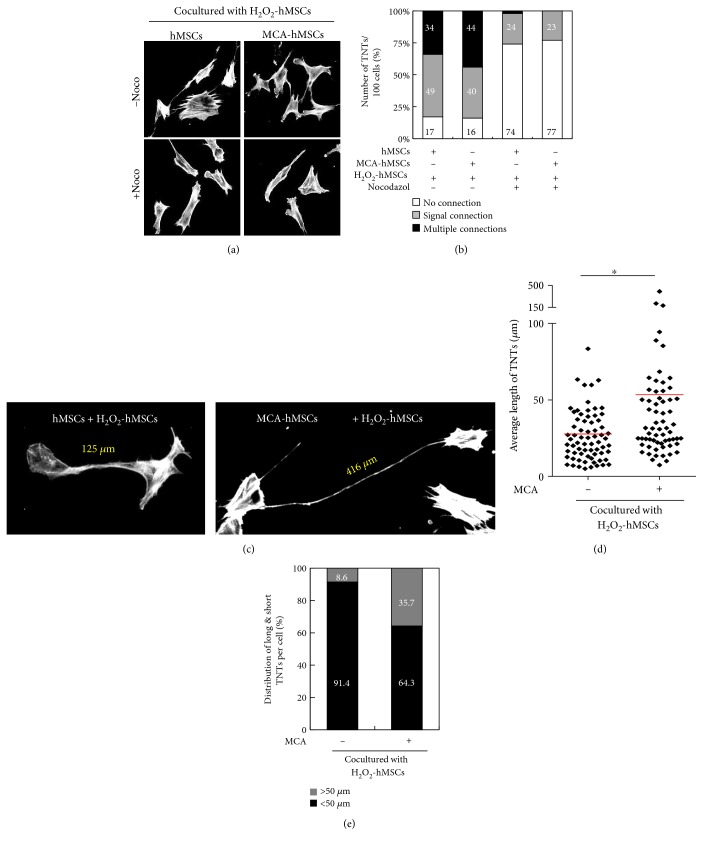
MCA enhanced TNT formation after coculture. TNTs decreased when microtubule-disrupting agent nocodazole (50 *μ*M) was included in the coculture system (a). The number of TNTs were enumerated and classified per 100 cells, and the proportion of TNTs that connected multiple cells increased from 34% to 44% after MCA treatment (b). Nocodazole almost disrupted multiple connected-TNTs in the coculture system. A representative image of long TNT after MCA treatment in the coculture system is shown in (c). The average length of TNTs formed in coculture containing H_2_O_2_-treated hMSCs and hMSCs with or without MCA were 53.5 *μ*m and 28.5 *μ*m, respectively (d). TNTs that were more than 50 *μ*m in length was 35.7% in the coculture containing MCA-treated and H_2_O_2_-treated hMSCs as compared to only 8.6% in the non-MCA-treated hMSCs coculture (e). ^∗^*p* < 0.05, as compared to the non-MCA-treated hMSCs coculture. Scale bar: 20 *μ*m.

We next investigated whether the formation of TNTs between the donors and recipients could be enhanced by MCA treatment. Figures 3(a) and 3(b) clearly demonstrated that the numbers of TNTs increased significantly after MCA treatment, while they decreased dramatically when microtubule-disrupting agent nocodazole (50 *μ*M) was included in the medium during the coculture period. MCA treatment also led to an increased number of long TNTs (Figures 3(c) and 3(d)). The average lengths of TNTs were 53.5 *μ*m and 28.5 *μ*m in coculture containing MCA-treated and control hMSCs, respectively (Figure 3(d)). Moreover, the TNTs that were more than 50 *μ*m in length was 35.7% in the coculture containing MCA-treated hMSCs (Figure 3(e)). Interestingly, MCA also increased the numbers of TNTs that connected two or more cells when compared to the non-MCA-treated coculture (44% versus 34%, Figure 3(b)).

### 3.3. MCA Enhances Mitochondrial Transfer

**Figure 4:**
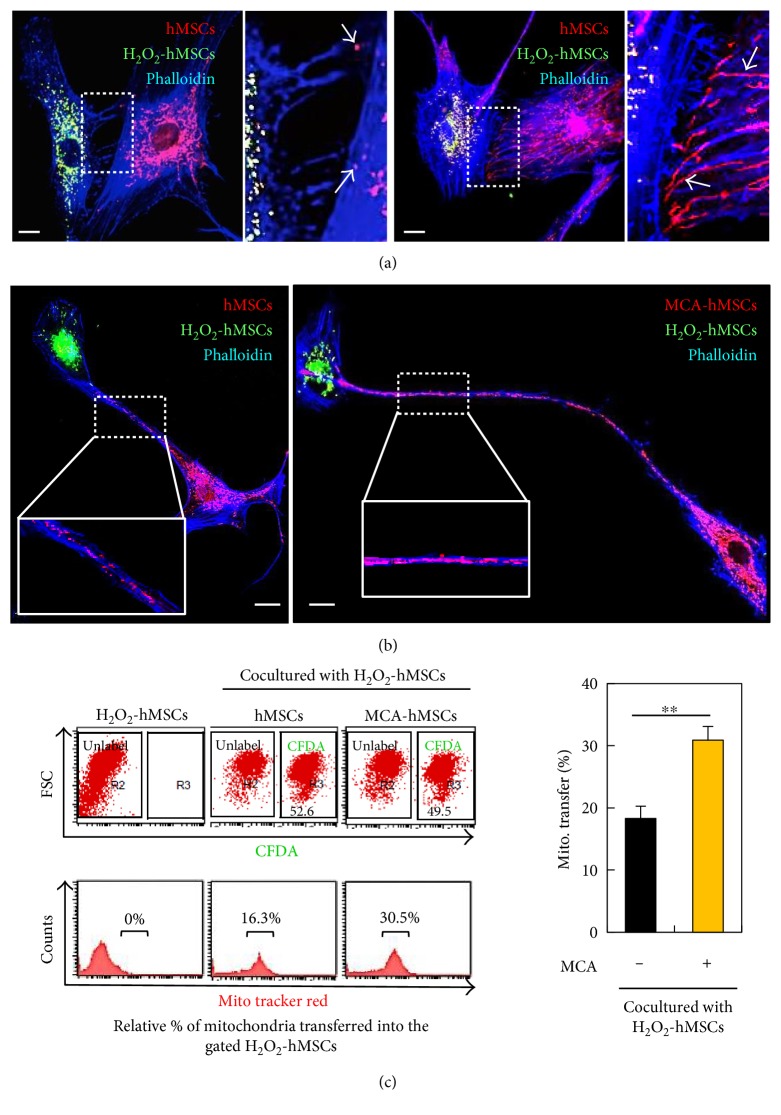
MCA enhanced mitochondria transfer to the injured hMSCs. (a) The MitoTracker Red-labeled mitochondria from the MCA-treated hMSCs were longer than the non-MCA-treated hMSCs in the coculture, as well as the numbers of TNTs formed (see enlarged insets). The actin was stained with phalloidin (blue fluorescence) in both coculture. (b) The MitoTracker Red-labeled mitochondria that resided along the TNTs were also longer in coculture containing MCA-treated hMSCs (right panel and inset) as compared to the one with non MCA-treated hMSCs (left panel and inset). (c) Healthy hMSCs with or without MCA treatment were labeled with CFDA and MitoTracker Red before coculture. After 24 h coculture with H_2_O_2_-treated hMSCs, all cells were harvested and subjected to flow cytometric analysis. The CFDA-unlabeled cells were gated and the MitoTracker Red fluorescence was quantitated. The proportion of cells containing red fluorescence was 30.5% in coculture containing MCA-treated hMSCs as compared to the 16.3% in the control hMSCs coculture. ^∗∗^*p* < 0.01.

We also observed not only that MCA increased the number and length of TNTs (Figure 3) but also that the morphology of the mitochondria was different between the MCA-treated and control cells. In Figures 4(a) and 4(b), the MitoTracker Red-labeled mitochondria from the MCA-treated hMSCs were much longer than those from the control. Interestingly, the mitochondria that resided along the TNTs were predominantly labeled with MitoTracker Red (Figure 4(b), also in Figure 2(a)). In order to make sure that the mitochondria were transferred or originated from the healthy hMSCs, we labeled the healthy hMSCs with or without MCA-treatment with MitoTracker Red (for mitochondria labeling) and CFDA (for cell labeling) and cocultured these cells with H_2_O_2_-treated hMSCs. After 24 h incubation, the total cells were harvested and subjected to flow cytometric analysis. The CFDA-free cells, that is, the H_2_O_2_-treated hMSCs, were gated and the fluorescence of MitoTracker Red was measured. The transfer of the MitoTracker Red-mitochondria from the MCA-treated cells significantly increased when compared to the control (30.5% versus 16.3%, Figure 4(c), right panel).

### 3.4. MCA-hMSCs Rescue Injured Cells by Reducing Oxidative Stress

**Figure 5:**
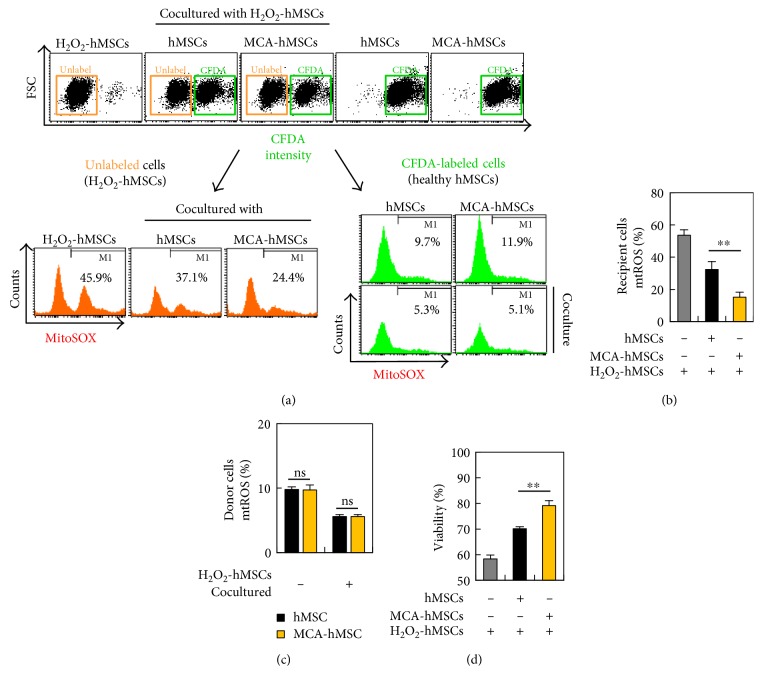
MCA-hMSCs rescued injured cells by reducing oxidative stress. (a) Healthy hMSCs with or without MCA treatment were labeled with CFDA before coculture. After 24 h coculture with H_2_O_2_-treated hMSCs, all cells were harvested, stained with and MitoSOX Red, and subjected to flow cytometric analysis. The florescence of MitoSOX Red was determined separately in the CFDA-labeled and CFDA-unlabeled cells. The proportion of injured cells (CFDA-unlabeled) with MitoSOX Red in coculture containing MCA-treated hMSCs was 24.4% as compared to the 37.1% in the control hMSCs coculture (b). However, the proportion of MitoSOX Red cells in the CFDA-labeled healthy cells did not differ much (c). The viability of injured cells increased after coculture (d). ^∗∗^*p* < 0.01, as compared with the control.

We next determined whether this mitochondrial transfer/donation was associated with reduction of oxidative stress. Oxidative stress was assessed by measuring mitochondrial ROS using MitoSOX Red (Figure 5(a)). In this experiment, the healthy cells were labeled with CFDA before coculture. After 24 h incubation, all cells were harvested, stained with MitoSOX Red, and subjected to flow cytometric analysis. Coculture with MCA-treated hMSCs markedly decreased the ROS level in the mitochondria of the recipient cells as compared to that of the control hMSCs (Figure 5(b), 32.4% versus 15.2%), whereas the ROS level in the donors' mitochondria (with or without MCA treatment) did not differ much (Figure 5(c)). Most importantly, the viability of the recipients also increased significantly if they were cocultured with healthy hMSCs (Figure 5(d), 79.2% and 71.7% in MCA-treated and control hMSCs, respectively).

### 3.5. Maintenance of Δ*Ψ*m after Coculture with MCA-hMSCs

**Figure 6:**
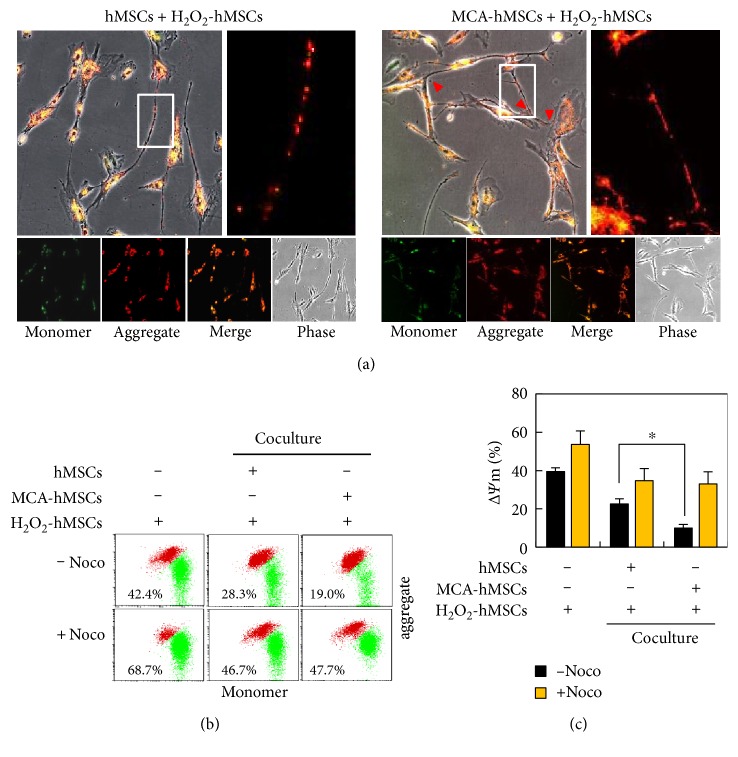
Maintenance of mitochondrial membrane potential (Δ*Ψ*m) after coculture with MCA-hMSCs. (a) The Δ*Ψ*m was measured using JC-1 in hMSCs coculture system. The JC-1 was mostly red (indicating higher Δ*Ψ*m) in coculture containing MCA-treated hMSCs (right panel), and the mitochondria appeared to be longer as compared to the control hMSCs coculture (comparing the enlarged insets from the right and left panels). (b) The cocultured cells were analyzed with flow cytometry. The proportion of cells with lower Δ*Ψ*m (green fluorescence) decreased in coculture containing MCA-treated hMSCs. The rescue of Δ*Ψ*m in the coculture decreased significantly in coculture medium containing nocodazole (c). ^∗^ *p* < 0.05.

In addition to reducing mitochondrial ROS in the recipients, we further demonstrated that this mitochondria transfer/donation also contributed to the maintenance of Δ*Ψ*m. The cocultured cells were stained with JC-1 and subjected to confocal microscopy. As shown in Figure 6(a), mitochondria with higher Δ*Ψ*m (red fluorescent) were dominant. The red and green fluorescence were further quantitated by flow cytometry, and the results demonstrated that the mitochondria with lower Δ*Ψ*m were significantly reduced in coculture containing healthy hMSCs (Figures 6(b) and 6(c)). The reduction was further enhanced in coculture containing MCA-treated hMSCs (Figures 6(b) and 6(c)). Addition of nocodazole significantly reduced this mitochondrial rescue effect, indicating that TNT formation was the key component in mitochondria transfer/donation (Figures 6(b) and 6(c)).

### 3.6. MCA Treatment Reduces Mitochondrial Fragmentation and Drp1 S616 Phosphorylation

**Figure 7:**
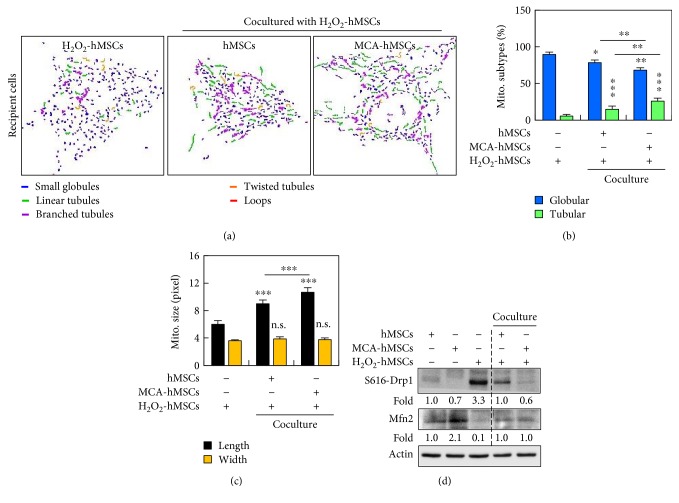
MCA reduced mitochondrial fragmentation. (a) Representative images of mitochondrial morphology after cocultured. The mitochondria can be classified into small globular, linear tubular, branched tubular, twisted tubular, and looped types, using MicroP software. The two major types of mitochondria, the globular and tubular, were enumerated and the results were shown in (b). In coculture containing MCA-treated hMSCs, the proportion of globular mitochondria decreased, while the proportion of the healthier tubular mitochondria increased significantly. (c) The length of the mitochondria in the coculture containing MCA-treated hMSCs was longer than that in the control hMSCs coculture. The width of the mitochondria from both coculture did not differ. (d) Western blot analysis of Drp1 S616 and Mfn2. The fold ratios of lanes 2 and 3 were obtained after comparing to lane 1, while that of lane 5 were obtained after comparing to lane 4. ^∗^*p* < 0.05, ^∗∗^*p* < 0.01, ^∗∗∗^*p* < 0.001, n.s., not significant. Scale bar: 20 *μ*m.

We nest investigated whether the protection of cell against oxidative damage, reduction of mitochondrial ROS, and maintenance of Δ*Ψ*m were reflected in mitochondrial dynamics. By using MicroP analysis, we quantitated the mitochondrial morphological changes after coculture. H_2_O_2_ caused fragmentation of almost 90% of mitochondria in the hMSCs (Figures 7(a) and 7(b)). However, coculture with healthy hMSCs reduced the proportion of globular mitochondria significantly (Figure 7(b)). Among them, coculture containing MCA-treated cells further reduced mitochondrial fragmentation to 68.1% when compared to the control hMSCs (78.5%) (Figure 7(b)). Concurrently, the proportion of the relative healthier linear tubular mitochondria consisted 14.8% and 26.0% in coculture containing control and MCA-treated hMSCs, respectively. In addition, the average length of the mitochondria in coculture containing MCA-treated hMSCs also significantly outscored those without MCA treatment or H_2_O_2_-monotreated cells (Figure 7(c)).

Recruitment and/or retention of Drp1 in the mitochondria have been implicated in mitochondrial dynamics [18, 19]. The polymerization of Drp1 is an early and obligatory step for mitochondrial fission and fragmentation. We next tried to explore the possible mechanism of how MCA reduced mitochondrial fragmentation in hMSCs by monitoring Drp1 phosphorylation (Drp1 S616) and Mfn2 expression. As shown in Figure 7(d), Western blot analysis revealed that exposure of hMSCs to H_2_O_2_ induced an increase in mitochondrial Drp1 S616, while Drp1 S616 was decreased in MCA-treated hMSCs, as well as in the coculture system. Notably, although MCA treatment increased Mfn2 expression in hMSCs, it did not alter the total expression of Mfn2 after coculture.

### 3.7. Mitochondria Transfer/Donation Helps in Maintaining Mitochondrion Quality in the Injured hMSCs

**Figure 8:**
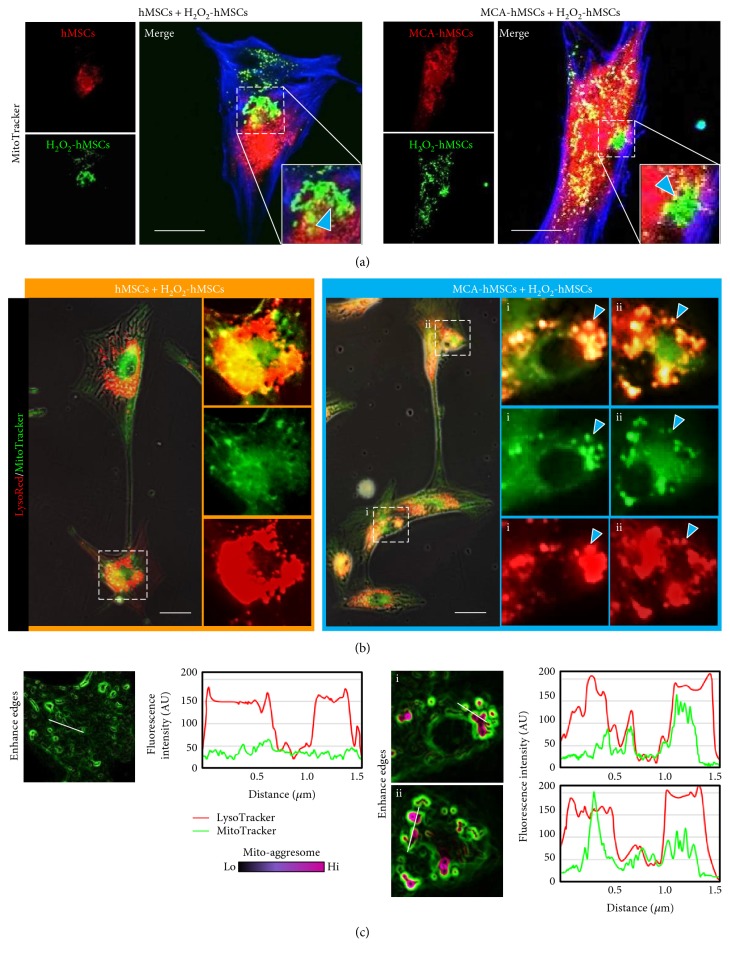
Mitochondria transfer helped regulating mitochondria quality in the injured hMSCs. (a) The mitochondria from the H_2_O_2_-hMSCs were labeled with MitoTracker Green, while the mitochondria from the healthy hMSCs were labeled with MitoTracker Red, respectively, before 6 h coculture. The number of MitoTracker Red-labeled mitochondria in the recipients increased in coculture containing MCA-treated hMSCs, while the MitoTracker Green-labeled mitochondria were shorter and tended to form aggregates (arrowheads in the enlarge insets). (b) Both cocultured cells were stained with MitoTracker Green and LysoRed to identify the fate of the short mitochondria. Many mitochondrial aggregates in the coculture containing MCA-treated hMSCs colocalized with LysoRed-labeled lysosomes (arrowheads in the enlarged insets of the right panel). (c) Fluorescence intensity analysis of the respective insets in (b) confirmed that the green fluorescence was in the same plane of the red fluorescence. Scale bar: 20 *μ*m.

Besides affecting mitochondrial dynamics by regulating Drp1 S616, we next investigated the fate of the damaged mitochondria in the H_2_O_2_-treated hMSCs after coculture with MCA-treated cells. MitoTracker Red and Green were used to label mitochondria in the healthy and H_2_O_2_-treated cells, respectively, before coculture. At 6 h after coculture, the total cells were subjected to fluorescent confocal microscopy. Numerous tubular intact mitochondria showed strong red fluorescence, while green fluorescence was found majorly in dots and short tubules (Figure 8(a)). Some of these dots and short tubules also harbored MitoTracker Red when the images were merged (insets in Figure 8(a)). The numbers of red fluorescent mitochondria also increased drastically in hMSCs pretreated with MCA (Figure 8(a), right panel), echoing the finding that MCA could increase mitochondrial mass in Figure 1(c).

We next investigated the fate of these dotted and short tubular mitochondria at 6 h after coculture with healthy hMSCs. The whole cells were labeled with MitoTracker Green and CytoPainter LysoRed. CytoPainter LysoRed selectively accumulates in lysosomes probably via the lysosome pH gradient and can be used to study autophagy, including mitophagy. Interestingly, some of the green fluorescent mitochondrial aggregates colocalized with LysoRed after we merged the images (Figure 8(b)). The colocalization of both cellular components was more prominently found in coculture that consisted of H_2_O_2_-treated and MCA-treated hMSCs (Figure 8(b), and the enlarged insets at the right panel). Fluorescence intensity analysis of the insets in Figure 8(b), especially in the two insets from MCA-hMSCs + H_2_O_2_-hMSCs, further confirmed that the green fluorescence was in the same plane of the red fluorescence (Figure 8(c)). The colocalization of fragmented mitochondria and lysosome could be a consequence of mitophagy, a mechanism that has been implicated in mitochondrial quality maintenance [20].

## 4. Discussion

Stem cell therapy is an important measure to restore tissue damage due to injury and might serve as an alternative to tissue or organ transplantation [21]. Besides successful application of hematopoietic stem cell transplantation in blood disorders or cancer patients, the therapeutic application of stem cells from other sources are not fully tested in human due to lack of exact mechanisms, poor viability of transplanted cells at the site of injury, poor integration of implanted cells with the damaged tissues, and ethical consideration [22]. Stem cells of other sources include embryonic stem cells, adult stem cells, and tissue/organ-specific progenitor cells. Among them, adult stem cells, especially tissue-derived MSCs, have attracted the interest of most researchers because they can be easily obtained from the bone marrow, adipose tissue, synovium, periosteum, and placenta without much ethical consideration [23]. However, the lack of exact mechanism and problem of low viability after transplantation remained unsolved. When MSCs enter the injury site, they usually die because of the harsh microenvironment and failure to integrate/adhere to the remaining tissue. In order to overcome the abovementioned problems, especially in hMSCs-based cell therapy, many strategies have been developed [22]. Genetic modification of MSCs before transplantation is less practical due to regulatory obstacles of gene therapy. Preconditioning of MSCs with chemicals, bioactive factors, and even hypoxia culture [24–26] seems much easier to approach. Furthermore, MSCs can be transplanted in allogeneic ways as they are less immunogenic [27].

Our previous studies show that NAC and AAP (termed MCA in current study) cotreatment could promote hMSCs entrance into S-phase by suppressing cyclin-dependent kinase inhibitors, which resulted in cell proliferation and yet retained their differentiation ability [15]. In addition, MCA also helped hMSCs to cope with H_2_O_2_-induced cell death by inhibiting cell death and maintaining mitochondria integrity [16].

**Figure 9:**
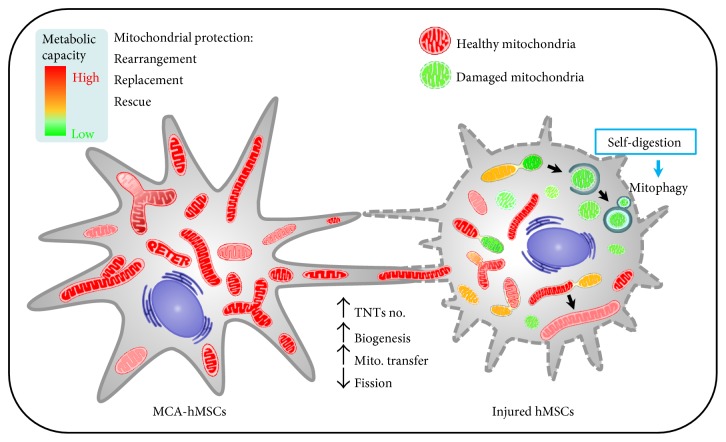
A schematic diagram showing TNT formation between injury and MCA-treated hMSCs. MCA can energize mitochondria and increase the number of TNTs in hMSCs. These healthy mitochondria can be transferred to the injured cells via TNTs and subsequently ameliorate oxidative stress, decrease mitochondrial damage/fragmentation/fission, and increase the turnover of damaged mitochondria by mitophagy.

In this study, using a coculture system that consisted of healthy and H_2_O_2_-trerated hMSCs, we demonstrated that MCA enhanced the protection potential of healthy hMSCs by mitochondria transfer/donation through TNTs. MCA initially promoted the increase of mitochondrial mass, respiratory capacity, and TNT formation. These “energized” mitochondria were transferred to the recipients via TNTs and decreased the oxidative stress of the H_2_O_2_-treated hMSCs. The Δ*Ψ*m of the injured cells was also stabilized after mitochondria transfer/donation. Concurrently, we also observed enhanced mitophagy in the coculture system, implicating that damaged mitochondria were being eliminated in order to maintain cell physiology (Figure 9). Although our findings indicate that functional mitochondria transfer/donation from MCA-treated hMSCs protects injured cells against H_2_O_2_-induced mitochondrial damage, the mechanisms of mitochondria transfer/donation remain unclear. Mechanistically, although the total Mfn2 did not increase after coculture, the decrease of Drp1 S616 was evidenced. Mfn2 and Drp1 are important regulators of mitochondrial fusion and fission, respectively.

Jiang et al. [28] demonstrated that human iPS-derived MSCs could also donate their functional mitochondria to rescue corneal epithelial cells under rotenone-induced oxidative stress via increased TNT formation between the two cell types. They further showed that the increased TNTs was associated with oxidative inflammation-activated NF*κ*B/TNF*α*ip2 signaling pathways that could be attenuated by NAC. Recent study has also revealed the possible mechanism of how mitochondria were transported in the TNTs: overexpression of Miro1, a mitochondrial Rho-GTPase, in mouse MSCs leads to enhanced mitochondrial transfer and rescue of epithelial injury, while knockdown of Miro1 in MSCs failed to do so [29]. Interestingly, Miro1 overexpression did not alter the anti-inflammatory factors of the MSCs, thus excluding the paracrine effects of MSCs in stem cell therapy.

## 5. Conclusion

This study reveals the protective effects of MCA in promoting the therapeutic potential of hMSCs by energizing mitochondria and enhancing mitochondria transfer/donation via TNTs (Figure 9). The pretreatment of hMSCs with antioxidants (MCA in current study) thus provides a novel strategy to enhance the therapeutic outcome of stem cell-based therapy in tissue injury.
